# Heat Stress After Pollination Reduces Kernel Number in Maize by Insufficient Assimilates

**DOI:** 10.3389/fgene.2021.728166

**Published:** 2021-10-08

**Authors:** Shiduo Niu, Xiong Du, Dejie Wei, Shanshan Liu, Qian Tang, Dahong Bian, Yarong Zhang, Yanhong Cui, Zhen Gao

**Affiliations:** College of Agronomy, Hebei Agricultural University/ State Key Laboratory of North China Crop Improvement and Regulation/ Key Laboratory of Crop Growth Regulation of Hebei Province, Baoding, China

**Keywords:** heat stress, maize, kernel abortion, assimilates, trehalose

## Abstract

Global warming has increased the occurrence of high temperature stress in plants, including maize, resulting in decreased the grain number and yield. Previous studies indicate that heat stress mainly damages the pollen grains and thus lowered maize grain number. Other field studies have shown that heat stress after pollination results in kernel abortion. However, the mechanism by which high temperature affect grain abortion following pollination remains unclear. Hence, this study investigated the field grown heat-resistant maize variety “Zhengdan 958” (ZD958) and heat-sensitive variety “Xianyu 335” (XY335) under a seven-day heat stress treatment (HT) after pollination. Under HT, the grain numbers of XY335 and ZD958 were reduced by 10.9% (*p =* 0.006) and 5.3% (*p =* 0.129), respectively. The RNA sequencing analysis showed a higher number of differentially expressed genes (DEGs) between HT and the control in XY335 compared to ZD958. Ribulose diphosphate carboxylase (RuBPCase) genes were downregulated by heat stress, and RuBPCase activity was significantly lowered by 14.1% (*p =* 0.020) in XY335 and 5.3% (*p =* 0.436) in ZD958 in comparison to CK. The soluble sugar and starch contents in the grains of XY335 were obviously reduced by 26.1 and 58.5%, respectively, with no distinct change observed in ZD958. Heat stress also inhibited the synthesis of grain starch, as shown by the low activities of metabolism-related enzymes. Under HT, the expression of trehalose metabolism genes in XY335 were upregulated, and these genes may be involved in kernel abortion at high temperature. In conclusion, this study revealed that post-pollination heat stress in maize mainly resulted in reduced carbohydrate availability for grain development, though the heat-resistant ZD958 was nevertheless able to maintain growth.

## Introduction

The rising levels of carbon dioxide in the atmosphere causes a greenhouse effect that results in increased temperatures and climatic changes (Xuan et al., 2020). Rising temperature is a global issue because of its impact on crop growth and yield (Wang et al., 2020a). Maize is more sensitive to heat stress (one of the most important abiotic stresses) than wheat and rice (Zhao et al., 2017; Zhang et al., 2019). Simulation result indicated that a 10% reduction in maize yield was shown for each 1°C increase in global temperature (Zhang and Zhao, 2017; Dong et al., 2021). Furthermore, previous studies have underscored the effects of high temperature on maize growth and development (Obaid et al., 2016; Zhang and Zhao, 2017; Wang et al., 2020b). For example, maize plants exhibited various effects of high temperature at distinct phenological periods (Lizaso et al., 2018). Maize tassels (male flowers) growing at the top of the plant were found to be vulnerable to low-level heat stress and this affected pollen viability (Dong et al., 2021; Wang et al., 2021). At the flowering stage, high temperatures inhibited anther dehiscence, pollen viability, and pollen germination, which caused kernel abortion and maize yield reduction (Carberry et al., 1989; Bakhtavar et al., 2015; Hatfield and Prueger, 2015; Li and Howell, 2021; Wang et al., 2021). Additionally, high temperatures caused a delay in the anthesis-silking interval (ASI) of maize, resulting in reduced kernel number, although plenty of pollen was still present (Wang et al., 2019). Moreover, in widely planted smaller tassel maize varieties, extended ASI distinctly decreased yield (Wang et al., 2019). Pollen sterility under heat stress has been intensely studied recently, but the manner in which high temperatures after pollination causes grain abortion remains unclear.

Leaf photosynthesis is fairly sensitive to high temperatures (Berry and Bjorkman, 1980), resulting in decrease in the net photosynthetic rate (Ben-Asher et al., 2008). Impaired photosynthesis affects biological carbon fixation (Gustin et al., 2018), thus restraining the synthesis of glucose and starch in the kernels and influencing the activities of related enzymes (Yang et al., 2016; Fahad et al., 2017; Basu et al., 2019). RNA sequencing (RNA-seq) analysis has shown that high temperatures downregulate starch synthesis genes involved in carbon metabolism (Bita and Gerats, 2013). Additionally, other stresses also have been shown to reduce assimilate availability, leading to kernel abortion (Puteh et al., 2014; Pan et al., 2015; Shen et al., 2020) and the inhibition of grain filling (Edreira and Otegui, 2012). Inversely, under abiotic stress, trehalose can increase sugar transport into the grains and improve crop grain number or grain size (Griffiths et al., 2016). Specifically, a gene in the maize ear expressing trehalose phosphate phosphatase causes a significantly reduced kernel abortion rate under drought (Nuccio et al., 2015). However, the effects of changes in sugar and trehalose-6-phosphate signaling synthesis genes on kernel abortion under short-term heat stress in field maize remains unclear.

Previous studies have shown that damage to the pollen grains due to high-temperature stress is the main limiting factor to kernel setting (Liu, 2014; Wang et al., 2019). Short-term heat stress after pollination was found to have less influence on maize kernel abortion (Wang et al., 2021). However, we hypothesized that post-pollination heat stress might result in kernel abortion in a heat-sensitive maize variety. Hence, the objectives of this study were to 1) assess the effects of heat stress on the change in kernel number after pollination using heat-sensitive and heat-resistant maize varieties and 2) determine sugar metabolism in the maize kernels under heat stress.

## Materials and Methods

### Experimental Site

The field experiment was conducted in 2019 at the Shenzhou Dryland Farming Experimental Station of the Hebei Academy of Agriculture and Forestry Sciences (Hebei Province, China, 37.91N, 115.71E). Supplementary Figure S1A shows the climatic conditions during the growing season of maize. The soil in the experiment was classified as loam fluvo-aquic with 12.53 g kg^−1^ organic matter, 65.8 mg kg^−1^ total nitrogen, 121.9 mg kg^−1^ available potassium, and 15.3 mg kg^−1^ available phosphorus.

### Experimental Design and Field Management

This study used the heat-sensitive “Xianyu 335” (XY335)and heat-insensitive maize varietiy: “Zhengdan 958” (ZD958) (Wang et al., 2020a). Both are common maize varieties in China. The maize seeds were manually sown at a density of 75,000 plants per hectare on June 16, 2019. The row spacing was 60 cm and the plant spacing was 22 cm and there were six rows in each greenhouse. After sowing, irrigation water was supplied using the surface flood method. The fertilizer application was done before sowing at a rate of 750 kg per hectare compound fertilizer with a 25:8:12 ratio of N: P_2_O_5_: K_2_O, while top-dressing was done at V12 using 138 kg N ha^−1^ (urea) of fertilizer. Weeds, pests, drought, and diseases were well controlled.

Randomized complete blocks were used in this study, with three replicates per treatment. The silking period was recorded when the silks of 50% of maize plants had reached 2 cm (Abendroth et al., 2011). Five days after silking, artificial unified pollination was conducted following Shen et al. (2020). Six simple greenhouses were then constructed to enclose the maize plants that would undergo heat treatment (HT), with each variety planted in a separate greenhouse. The control maize plants were grown under natural conditions. Each greenhouse measured 5 m in length, 3.5 m in width, and 3.5 m in height. Polyethylene film (0.8 mm thickness) was used as a barrier with 1.2 m openings on the sides for adequate gas exchange (Supplementary Figure S2). The HT treatment was conducted from 8:00 to 18:00 for 7 days.

Temperature and humidity recorders (L95-2 Saiouhuachuang Technological Corporation, Beijing, China) were installed in the center of each greenhouse to record data every 10 min and were placed 1.5 m above the ground. The average temperature and maximum temperature in the shed during daily treatment are shown in Supplementary Figure S1B. The simple greenhouses were removed after the HT treatment.

### Sampling and Measurements

Four days after HT, the net photosynthetic rate (Pn) of the ear leaves (representative source organ) was measured with a portable photosynthetic apparatus system (LI-3400 Li-Cor, USA) under a natural field environment. Each measurement was taken at the center of the ear-leaf.

Light intensity was measured at noon above the canopy three times in each plot by using a LI-250A Light Meter (Li-Cor, USA). The polyethylene film used for the HT allowed a penetration of 95.4% of the incoming solar radiation. There was no significant difference in light intensity between HT and the control (CK) (Supplementary Figure S1C).

At 5 days after the HT treatment (10 days after silking), sampling of the leaves and kernels was performed. Each leaf sample (>1 g) was taken from the bottom of the ear leaf at about 20 cm. The fresh kernel samples (>20 grains) were taken as shown in Supplementary Figure S3. Samples of the kernels and leaves were immediately frozen in liquid nitrogen. The samples were then refrigerated at −80°C for enzyme activities determination and transcriptome sequencing. At 10 days after silking, three ears per plot were gathered, and two rows of grains were sampled for dry weight measurements. The dry weight was determined by drying the kernels at 80°C until a constant weight. The ear growth rate of the maize was then calculated based on the dry weight of the ear (cob + kernel). At physiological maturity, the maize ears were hand-harvested in each treatment to record the row number and kernel number per row.

### Assay of Soluble Sugar and Starch Levels

The soluble sugar content of the kernels was measured using the anthrone colorimetric method. Briefly, 0.5 g of dried powdered of kernels was mixed with 6 ml of water and then heated at 100°C for 30 min. The samples were cooled to room temperature and centrifuged at 3,500 rpm for 15 min to obtain the supernatant as a soluble sugar solution. The supernatant was transferred into another test tube and the last procedure was repeated. The insoluble sediment was diluted with 10 ml of 3 mol L^−1^ HCl and then heated for 45 min at 100°C. Following which it was centrifuged at 35,000 rpm min^−1^ for starch determination. The product was collected and neutralized with 10 ml of 3 mol L^−1^ NaOH. The measurement of soluble sugar and starch levels referred to Hanft and Jones (1986).

### Measurement of Photosynthetic Enzyme and Starch Synthase Activities

The fresh kernel samples (0.5 g) were ground into a fine powder and extracted with 450 μL Phosphate Buffer Solution (PBS) at pH 7.2–7.4. Afterwards, the prepared samples were centrifuged at 4,000 rpm min^−1^ for 15 min to separate the supernatants, which were then assayed using an enzyme-linked immunosorbent assay (ELISA) kit (Sci-tech innovation, Qingdao, China). Activities of starch synthase (SSS), adenosine diphosphate-glucose pyro phosphorylase (AGPase), and cell wall invertase (CWIN), which are three key enzymes involved in the starch synthesis pathway in maize kernels, were determined. In addition, the ribulose diphosphate carboxylase (RuBPCase) activities were measured in the leaves were measured following Zhang et al. (2020a).

### Transcriptome Analysis

Transcriptome analysis was conducted on both the leaves and the kernels of the two maize varieties grown under unstressed (CK) and stressed (HT) conditions in a field environment. The total RNA was extracted using TRizol reagent (Invitrogen, Carlsbad, CA) following the manufacturer’s directions with three biological repeats tested. The RNA concentration, purity, and integrity were measured using a NanoDrop 2000 (Thermo Fisher Scientific, Wilmington, DE) and the RNA Nano 6000 Assay Kit of the Agilent Bioanalyzer 2100 system (Agilent Technologies, CA, USA), respectively. The input material for RNA sample preparations was 1 μg per sample. The database sequencing libraries were established following the manufacturer’s recommendations for the NEBNext® UltraTMRNA Library Prep Kit for Illumina® (NEB, USA). Index codes were added to attribute sequences to each sample. As instructed by the manufacturer, the clustering of the index-coded samples was performed on the cBot Cluster generation system through a TruSeq PE Cluster Kit v4-cBot-HS (Illumina). After cluster generation, the library preparations were sequenced on an Illumina platform and paired-end reads were generated. The sequence analysis was performed using the BMKCloud platform (www.biocloud.net). The data were subjected to strict quality control by deleting low-quality sequence reads. The data considered were reads with a proportion of N higher than 10%, and reads with a quality value of Q ≤ 10 accounted for more than 50% of the total reads. The clean data were mapped to the maize reference genome (B73_RefGen_v2) using HISAT2 (Kim et al., 2015). The gene expression outputs statistical data is given as follows Supplementary Table S1. After quality control of sequencing data, 189.04Gb Clean Data were obtained and the minimum of Q30 was 94.73%).

The mapped read numbers and transcript length were normalized. Fragments Per Kilobase of transcript per Million fragments mapped (FPKM; Florea et al., 2013) was used as an index for the gene expression levels in different samples.

The differentially expressed genes (DEGs) were selected based on log_2_ (fold change) >1 or log_2_ (fold change) <−1 and with statistical significance of *p <* 0.05. The transformed and normalized expression values of the DEGs FPKM by Z-score were used for hierarchical clustering. Supplementary Table S2 shows the annotations of the enzyme related genes described in this study. Supplementary Table S3 shows the related annotations of heat shock genes in this study. The annotations of genes involved in photosynthesis were sourced from the database of the National Biotechnology Information Center (NCBI, https://www.ncbi.nlm.nih.gov/).

### Quantitative Real Time-PCR

The RNA-seq data were further validated by quantifying the gene expression of a selected number of genes in the XY335 kernels using quantitative real-time PCR analysis (qRT-PCR). The cDNA synthesis from the total RNA was performed using the TRUEscript 1st Strand cDNA SYNTHESIS Kit (Aidlab, Beijing, China), and qRT-PCR are done with 5 × RT Reaction Mix (MedChemExpress, China). The specific primers used in the qRT-PCR are listed in Supplementary Table S4. The primers were designed based on gene sequences in the NCBI GenBank database and were synthesized by Biomarker Technologies (Beijing, China). The fluorescence was measured at the end of each cycle for quantification. Using GRMZM2G171060 (Zm00001d000379) as the reference gene, the 2^−ΔΔCt^ method was used to calculate relative gene expression with three technical replicates tested. The qRT-PCR results showed that the transcriptome results were reliable (Supplementary Figure S4).

### Statistical Analysis and Drawing of Illustrations

A two-tailed Student’s *t-test* was used to determine significance levels between the CK and HT in kernel number, ear growth rate, enzymatic activity, sugar content, and photosynthesis rate. Statistical analyses were performed using IBM SPSS Statistics Version 25 and Microsoft Excel 2019.

Figures were drawn using SigmaPlot 12.5 and Adobe Illustrator CC 2020. A heatmap of the DEGs was drawn using the R package Pheatmap. Statistics of pathway enrichment were drawn by the platform BMKCloud (http://www.biocloud.net).

## Results

### Kernel Number and Ear Growth Under Heat Stress

**FIGURE 1:**
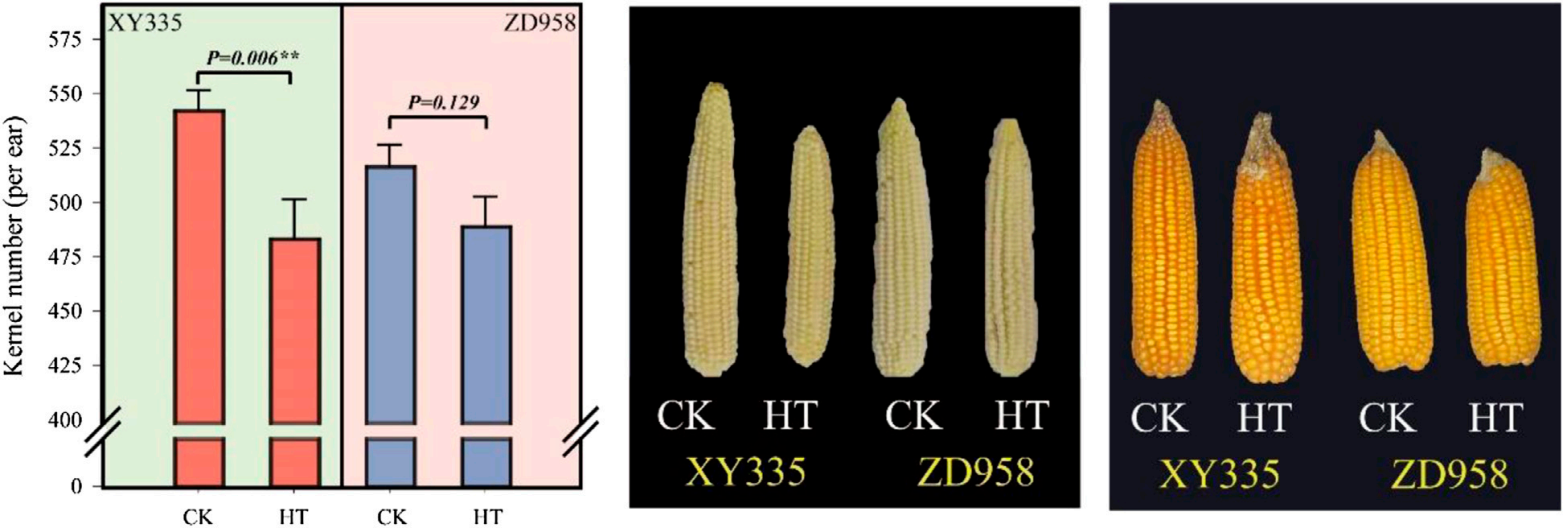
Effects of heat stress on the kernel number of maize varieties (ZD958 and XY335) grown under control (CK) and heat treatment (HT) conditions.

**FIGURE 2:**
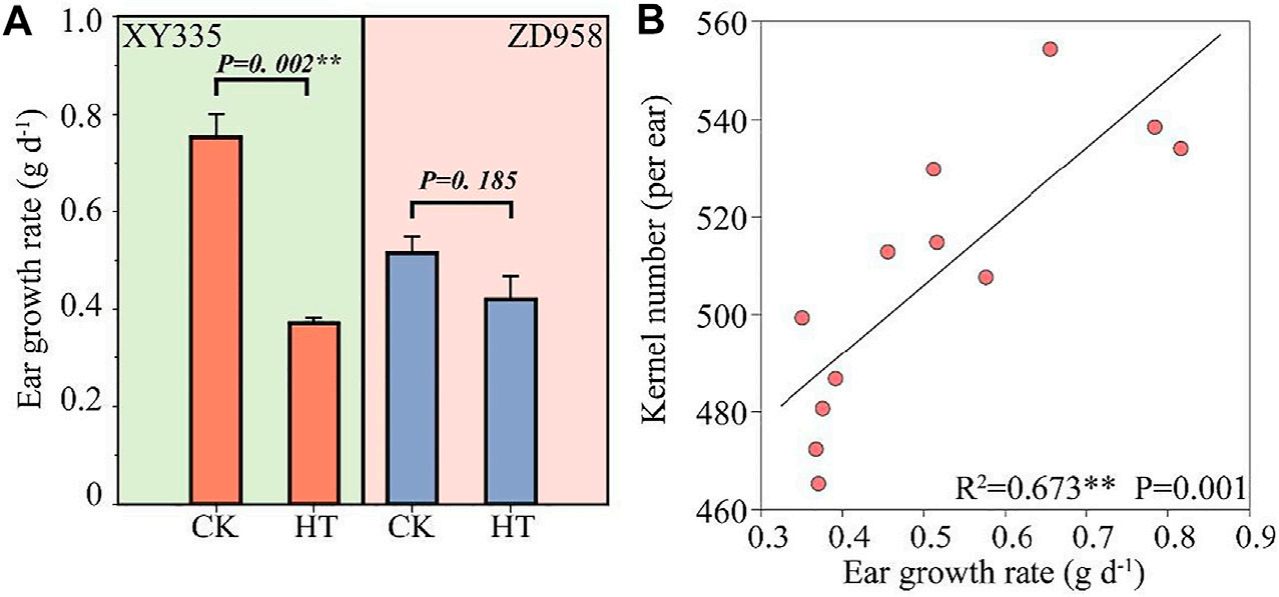
**(A)** Effects of heat stress on ear growth rate after 5 days of heat treatment. CK and HT indicate the control and heat treatment, respectively. **(B)** Relationship between the ear growth rate after 5 days of heat treatment and the kernel number per plant.

As shown in Figure 1, the kernel number per ear under HT in XY335 was reduced by 10.9% compared to CK, while no significant difference was observed between CK and HT in ZD958. The ear growth rate significantly decreased under high temperatures. The ear growth rate of XY335 and ZD958 under HT was significantly reduced by 50.6 and 18.4% compared to CK, respectively (Figure 2A). Moreover, the correlation analysis results showed that high ear growth rate around pollination increased the kernel number (*p =* 0.001, Figure 2B).

### Analysis of Gene Expression and Differentially Expressed Genes

**FIGURE 3:**
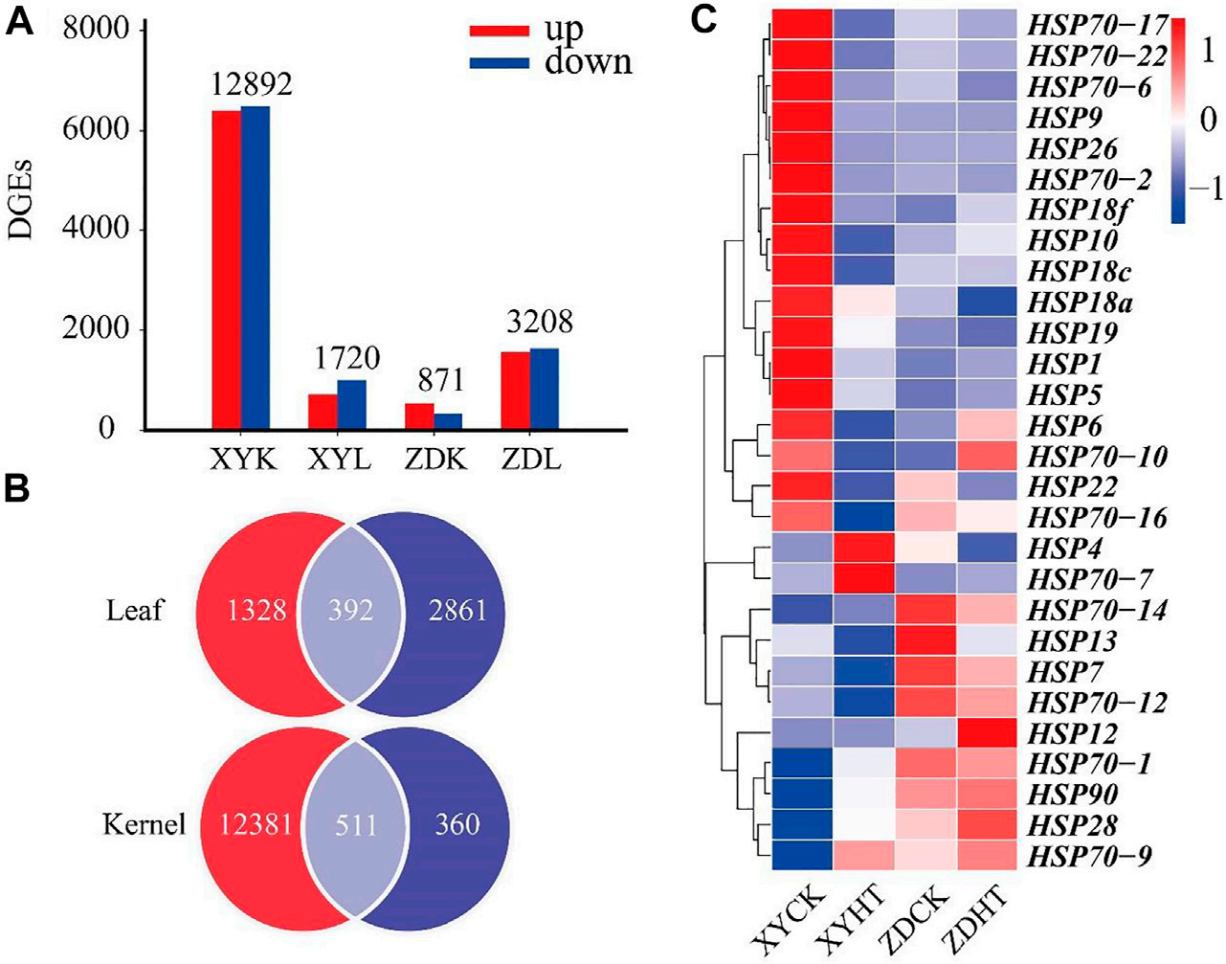
Comparing the differentially expressed genes (DEGs) of maize varieties XY335 and ZD958 grown under both control (CK) and heat (HT) treatments on the 5th day of HT. **(A)** Total numbers of upregulated and downregulated genes **(B)** Venn diagram of the DGEs and **(C)** heatmap of the genes related to heat shock under heat stress.

Figure 3A shows the results of the transcriptome analyses of DEGs in the HT treatment compared to the CK. The leaf and kernel samples of XY335 had 871 and 12,891 DEGs, respectively. The numbers of DEGs identified in the leaf and kernel samples in ZD958 were 3,208 and 1,720, respectively. Under the same heat stress, the number of DEGs detected in ZD958 was considerably lower than in XY335, with 392 and 511 DGEs overlapping between the leaf and kernel, respectively (Figure 3B). The heatmap of heat shock genes showed 20 upregulated genes and five downregulated genes in XY335, while normal expression levels were detected for these genes in ZD958. Remarkably, HSP4 was downregulated in ZD958 but normal in expression XY335 (Figure 3C). Therefore, this study showed that XY335 was greatly affected by high temperature, whereas ZD958 was not.

### Effect of Heat Stress on Photosynthesis

**FIGURE 4:**
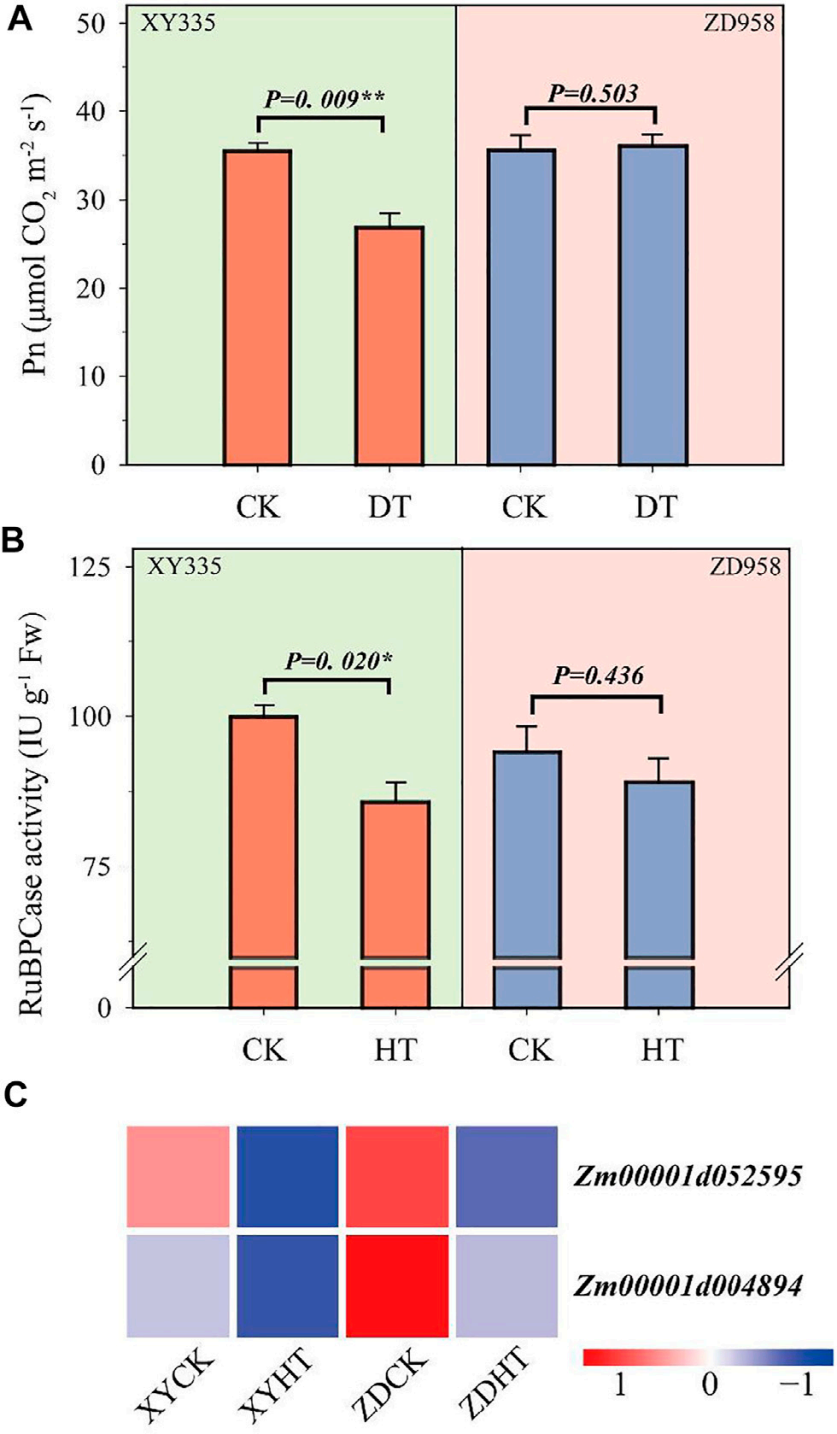
Changes in photosynthesis after 5 days of heat treatment (HT) compared to the control (CK): **(A)** photosynthesis rate in CK and HT; **(B)** ribulose diphosphatecarboxylase (RuBPCase) activity in CK and HT; and **(C)** heatmap of the genes related to RuBPCase under heat stress. Red and blue lines represent upregulated and downregulated genes, respectively.

The leaf net photosynthetic rate (Pn) of XY335 was significantly inhibited by heat stress, whereas the Pn of ZD958 was not obviously reduced (Figure 4A). Similarly, RuBPCase activity under HT was significantly decreased by 14.1% compared with CK in XY335. HT lowered the RuBPCase activity by 5.3% less than CK in ZD958 (Figure 4B).

A total of 183 genes related to photosynthesis were analyzed, and the two varieties showed different results under the CK and HT treatments (Supplementary Figure S5). Among these genes, 131 and 133 genes were downregulated in XY335 and ZD958, respectively. Additionally, 62 genes differed between the two varieties. Unexpectedly, two genes encoding RuBPCase, Zm00001d004894 and Zm00001d052595 were significantly downregulated in both XY335 and ZD958 under HT (Figure 4C).

### Sugar Metabolism in the Maize Kernels

**FIGURE 5:**
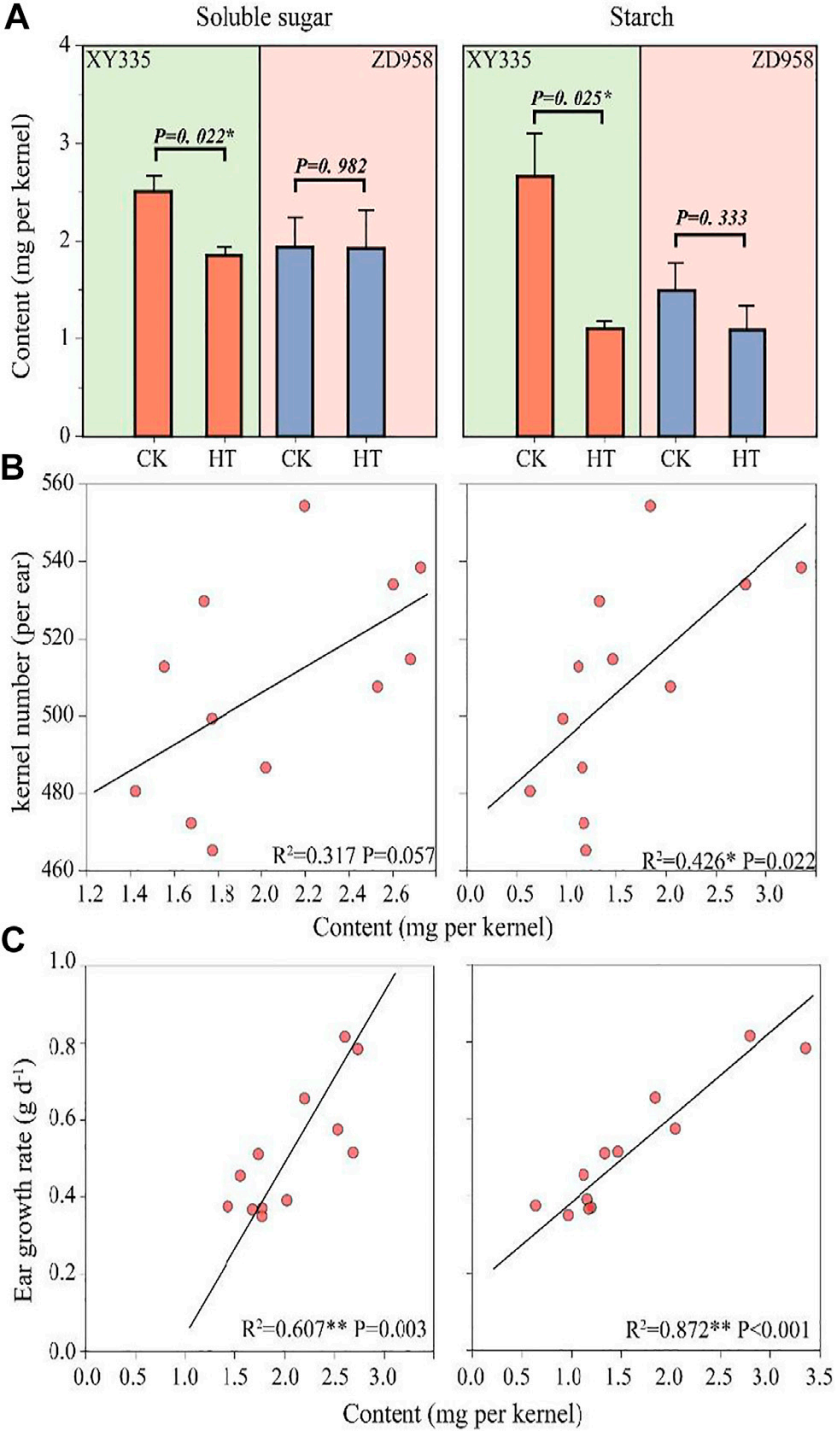
Effects of heat stress on soluble sugar, and starch content in the kernel and relationships between soluble sugar/starch with kernel number and ear growth rate. **(A)** Soluble sugar and starch contents in the maize kernels as affected by heat treatment (HT) after the 5th day of treatment. **(B)** The relationship between the soluble sugar (left)/starch (right) contents with final kernel number. **(C)** The relationships between the soluble sugar (left)/starch (right) contents with the ear growth rate.

The soluble sugar in the XY335 kernels under HT decreased by 26.1% compared with CK, while there was no remarkable reduction in soluble sugar content in ZD958 under HT. The kernel starch content in XY335 and ZD958 under HT decreased by 58.5 and 27.2% compared with those of CK, respectively. Figure 5A shows that the decrease in kernel starch contents reached a significant level (*p=* 0.025) in XY335 but not in ZD958 (*p=* 0.333). Correlation analysis indicated that sufficient soluble sugar (*p= 0.056*) and starch (*p=* 0.021) could increase kernel number (Figure 5B). Additionally, soluble sugar content (*p =* 0.003) and starch content (*p <* 0.001) were positively correlated with ear growth rate (Figure 5C).

**FIGURE 6:**
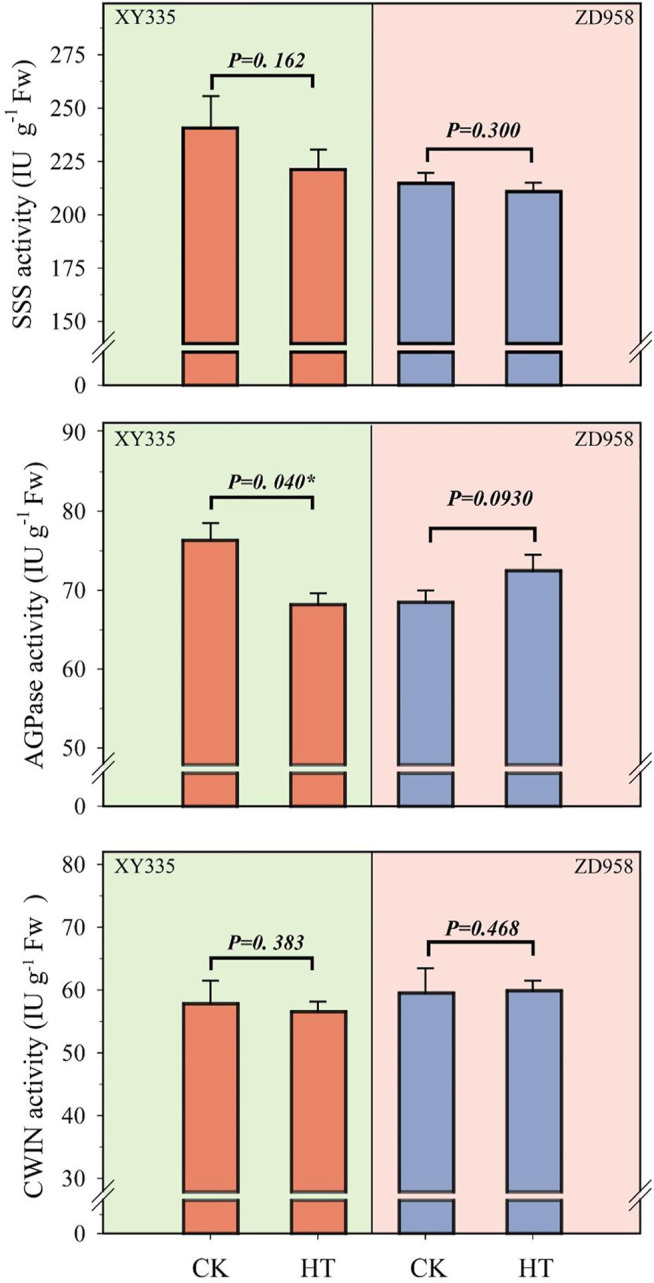
Changes in the activities of starch synthase (SSS), adenosine diphosphate-glucose pyrophosphorylase (AGPase), and cell wall invertase (CWIN) at 5 days after heat treatment.

**FIGURE 7:**
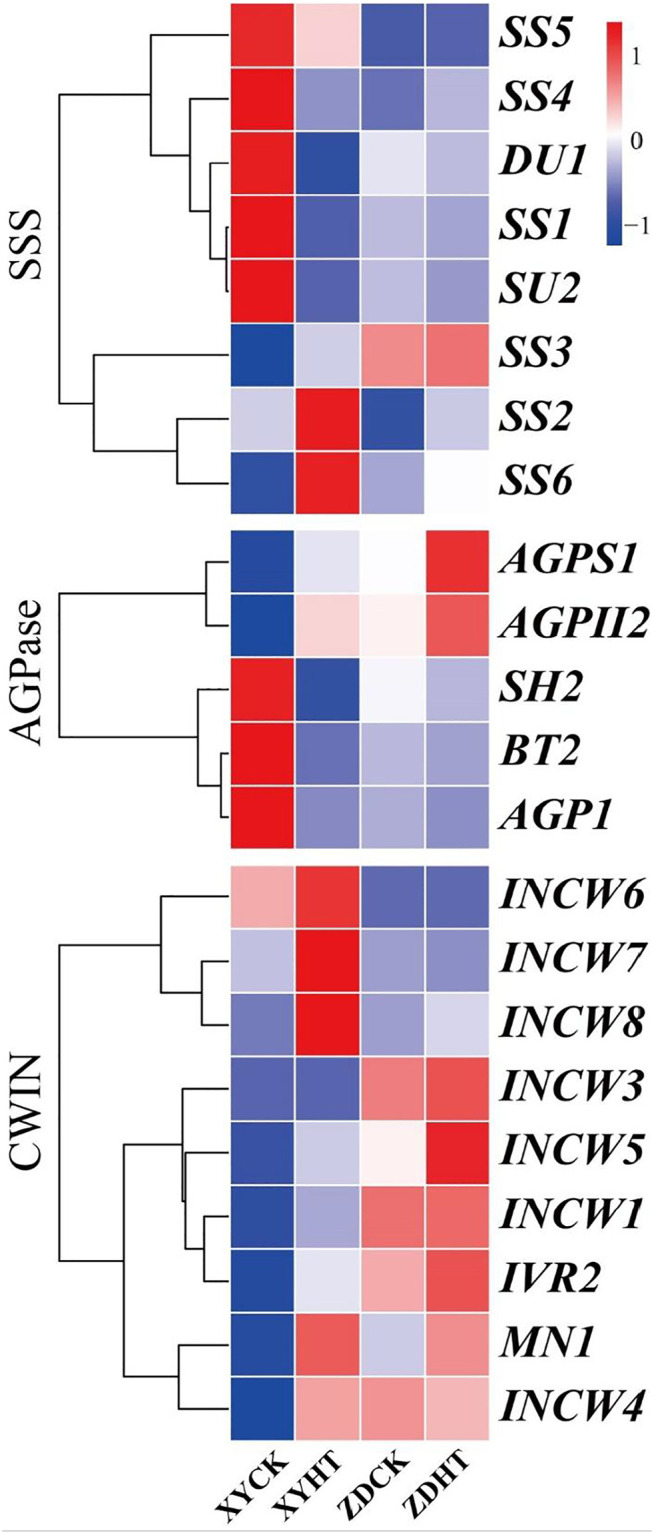
Expression profiles of genes that encode starch synthase (SSS), adenosine diphosphate-glucose pyrophosphorylase (ADGPase), and cell wall invertase (CWIN). **(A)** Gene expression (FPKM fold change) of the candidate genes in maize leaves under CK and HT conditions.

Under HT condition, the activities of CWIN and SSS in the kernel did not change significantly, while AGPase activity was found to be sensitive to HT treatment (Figure 6). The results showed that the SSS activity under HT was reduced by 8.1% (*p=* 0.162) and 1.8% (*p=* 0.300) in XY335 and ZD958, respectively. The AGPase activity in the maize kernels decreased significantly by 10.6% in XY335, while a 5.8% increase was observed in ZD958, though this was not statistically significant (*p=* 0.093). The RNA-seq results indicated that five SSS genes and three AGPase genes were downregulated under the HT relative to CK. Interestingly, CWIN-related genes exhibited upregulated expression compared to those in the CK group (Figure 7).

**FIGURE 8:**
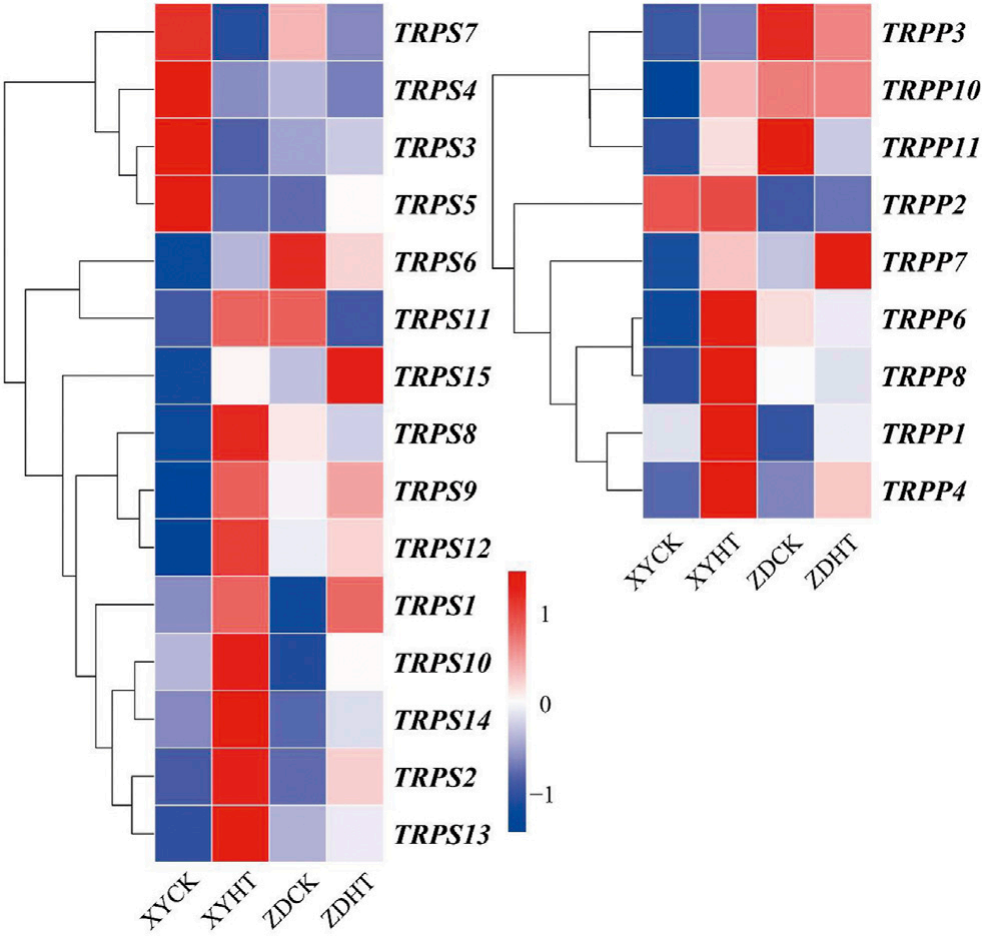
Variation in expression profiles of the genes involved in trehalose biosynthesis.

Additionally, 20 genes related to trehalose synthesis were upregulated, whereas four genes were downregulated. In the metabolic pathways of starch and sucrose, trehalose-synthesized genes were found to be upregulated in XY335 under HT; however, ZD958 showed relatively minor changes (Figure 8). From the results, it is clear that starch synthesis was inhibited, while trehalose synthesis was promoted under high temperature stress, which may have resulted in kernel abortion.

## Discussion

Previous studies have shown that maize pollens are susceptible to high temperature, usually resulting in kernel abortion (Wang et al., 2019; Wang et al., 2021). Our results indicated that heat stress after pollination still plays a critical role in kernel abortion, and it reduces carbohydrate availability and damages carbon metabolism. Additionally, kernel abortion after pollination at a high temperature was found to be variety-specific.

### Limited Low Ear Growth Rate in the Early Stage of Maize Grain Filling Reduced the Kernel Number

During the critical growth stage bracketing of silking, stress typically reduces the maize plant/ear growth rate (Rossini et al., 2011, Pagano and Maddonni, 2007; Borras and Vitantonio-Mazzini, 2018). Kernel number per ear is significantly related to plant growth rate around silking and biomass partitioning to the ear during this period (Pagano and Maddonni, 2007). In this experiment, heat stress also decreased maize ear growth rate, especially for XY335. HT after pollination reduced ear leaf photosynthesis and decreased assimilate availability, which reduced ear growth. Moreover, previous research has shown that the ear growth rate at the early stage of kernels growth determined the final grain number (Rossini et al., 2011). In line with previous studies, there was a significant correlation between the kernel number and the ear growth rate during this period in our experiment (Figure 2B). As discussed, the kernel number was affected by the restriction of ear growth rate under HT.

### Insufficient Sugar Supply Leads to Restricted Ear Growth Rate After Pollination

Maize is very sensitive to high temperatures during tasseling, flowering, pollination, and kernel filling (Zhang et al., 2020b). The reasons may be that 1) a high temperature causes kernel abortion by destroying pollination processes (Deryng et al., 2014) and 2) a high temperature causes sugar deficiency or insufficient sugar metabolism, leading to kernel abortion (Edreira and Otegui, 2012). A previous study showed that 22.1% of the kernels were aborted during the 15-days high temperature stress treatment after tasseling (Wang et al., 2021). The results of this study showed that 10.9% of kernels in the XY335 maize variety were aborted after post-pollination heat stress. This also suggested that the abortion of a large percentage of maize kernels can still occur post-pollination under high temperature conditions.

Significant decreases in the leaf photosynthetic rate as well as in soluble sugar and starch contents in the kernels were observed under high-temperature stress (Figures 4A, 5). The poor supply of sugars induces ovary abortion, ultimately affecting kernel formation and yield in maize (Gao et al., 2020; Usmani et al., 2020). Sucrose feeding can reverse the kernel loss induced by drought or shade stress (Hiyane et al., 2010; Zinselmeier et al., 1995) and can partially restore the activity of carbon metabolism-related enzymes, thus restoring some kernel growth (McLaughlin and Boyer, 2004). These findings imply that kernel abortion is associated with an insufficient assimilate supply (Puteh et al., 2014; Edreira and Otegui, 2012). Carbon-related enzymes also play a vital role in kernel abortion (Shen et al., 2020), as demonstrated in this study where kernel abortion was caused by the decreased activity of enzymes related to starch synthesis and reduced levels of starch (Figure 5B). Transcriptome analysis also showed that heat stress caused the low expression of starch synthesis-related genes. Overall, the results of this study are consistent with previous studies (Yue et al., 2016; Lambarey et al., 2020; Shen et al., 2020).

Interestingly, no significant change in the activity of CWIN was observed, but its related genes were upregulated. CWINs contribute to sink strength and have been previously reported to exhibit a key role in sucrose import and kernel filling (Millera and Chourey, 1992; Weber et al., 1995; Wang et al., 2008; Morey et al., 2018). We speculated that a low sugar supply promoted the upregulation of CWIN, but we found that heat stress inhibited CWIN activity and reduced sucrose import.

### The Trehalose Pathway Affected Kernel Setting Under High Temperature

Trehalose biosynthesis has been confirmed to increase tolerance to multiple abiotic stresses in tobacco, potato, and rice (Schluepmann and Paul, 2009). In plants, trehalose-6-phosphate synthase (TPS) catalyzes UDP-glucose and glucose-6-phosphate to synthesize trehalose-6-phosphate (T6P) (Kumar et al., 2013). T6P is further metabolized to trehalose by trehalose-6-phosphate phosphatase (TPP) (O’Hara et al., 2013). T6P is a critical signaling molecule that integrates sugar status with growth and development in plants (Paul et al., 2017; Gustin et al., 2018; Paul et al., 2020). The results of this study showed that under high temperatures, both the TPS and TPP genes were obviously upregulated in XY335, a heat-sensitive maize variety, while no obvious change was observed in ZD958, a heat-resistant variety. The results suggested that trehalose metabolism played an important role in kernel abortion. The similar changes may occur under shade stress (Liang et al., 2020). Additionally, a reduction in T6P level via the expression of TPP can prevent maize kernel abortion and increase yield under drought stress (Nuccio et al., 2015). The elevated gene expressions of TPS and lower gene expression of TPP in the apical kernels inhibited seed setting (Shen et al., 2020). In this study, both the TPS and TPP genes were upregulated in XY335 under heat stress, but relatively unobvious changes were observed in ZD958. This suggested that the response of trehalose metabolism to heat stress was distinct in the heat-sensitive variety, ultimately leading to kernel abortion under heat stress.

### ZD958 has Higher Heat Resistance Than XY335

XY335 is sensitive type to environmental stress, including heat stress whereas ZD958 is resistant to environmental stress (Liu, 2014; Wang et al., 2020a). The yield of the former is typically higher than that of the latter. However, yield performance differs markedly under stress (Berry and Bjorkman, 1980). In a stressful environment, low assimilate availability aggravates kernel abortion (Gao et al., 2020; Shen et al., 2018). However, sugar stored in the stems can serve as a buffer to ensure kernel growth (Milne et al., 2013; Bledsoe et al., 2017). This study showed that the heat-sensitive (XY335) had a higher kernel number than the heat-tolerant variety (ZD958) under control conditions. Under HT conditions, ZD958 produced more kernels compared to XY335. Although the sugar content in the stem was not measured in this study, all the data gathered, including the net photosynthetic rate, DEGs, and final kernel number, demonstrated that ZD958 is a heat-resistant variety.

**FIGURE 9:**
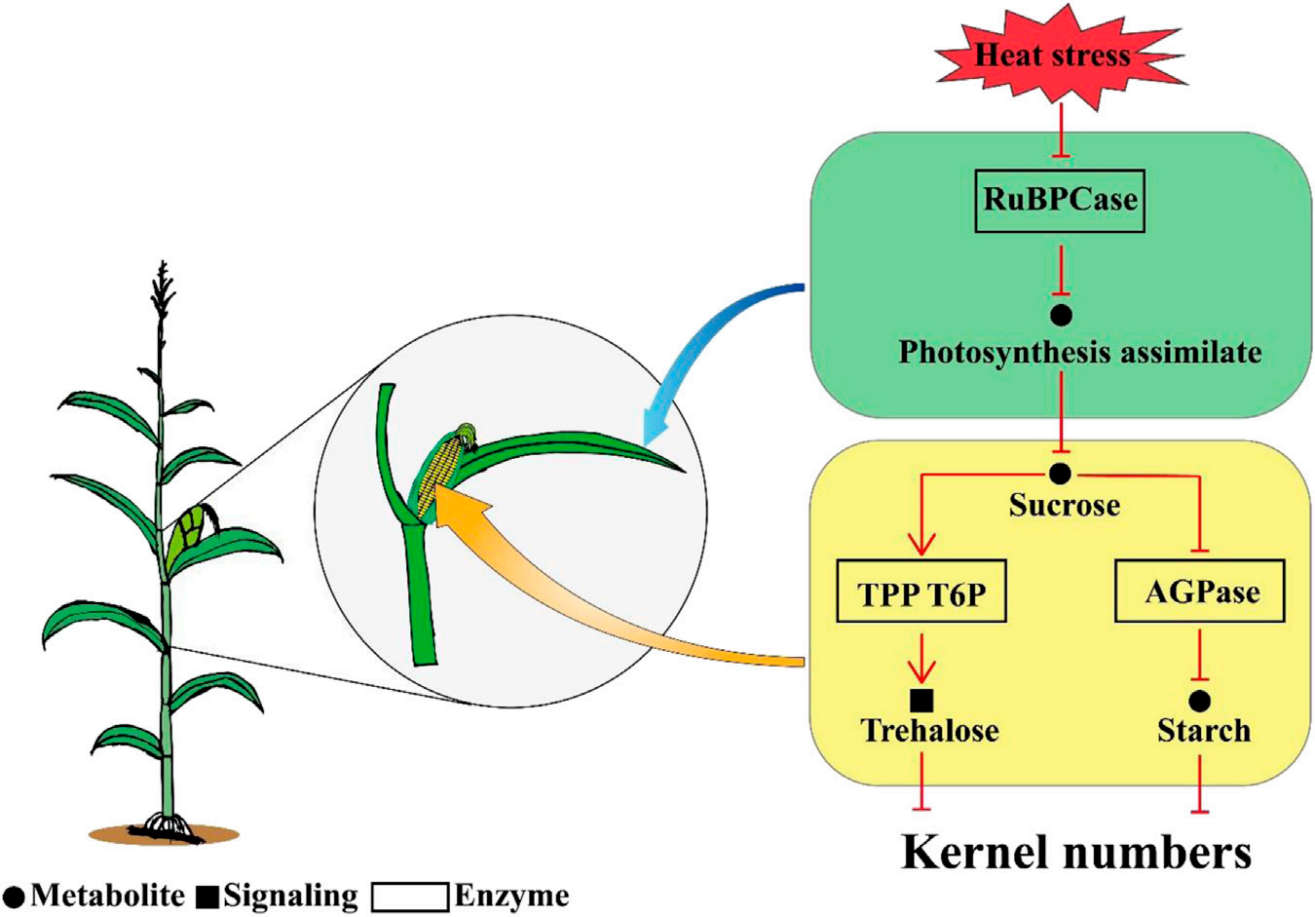
A schematic diagram model for the mechanism of kernel abortion, including transcripts, starch and sucrose metabolism and photosynthesis.

Overall, the results of this study indicated that kernel abortion was caused by carbohydrate metabolic disorders. Heat stress decreased the RuBPCase activity by downregulating Zm0001d052595 and Zm0001d004894 which restricted photosynthesis and decreased assimilate availability for the kernels. The downregulation of genes related to AGPase and the upregulation of genes related TPP resulted in T6P disrupting the balance between trehalose and starch. Consequently, this study demonstrates that reduced carbohydrate availability leads to kernel abortion under post-pollination heat stress conditions (Figure 9).

## Conclusion

Heat stress after pollination can result in kernel abortion, especially in heat sensitive varieties. Heat stress mainly reduces leaf photosynthesis and RuBPCase activity thus lowering assimilate availability. Ear growth rate was significantly reduced and showed significant relationship with kernel number. Concurrently, the soluble sugar and starch content and key enzyme activity in the kernels were decreased and the related genes also showed obvious downregulation. Additionally, the altered synthetic pathway of trehalose may play a critical role in kernel setting under heat stress. In conclusion, heat stress in maize after pollination results in kernel abortion due to insufficient assimilate availability.

## Data Availability

The datasets presented in this study can be found in online repositories. The names of the repository/repositories and accession number(s) can be found below: NCBI SRA; PRJNA757605.

## Supplementary Material

Supplementary Figure 1 The meteorological data during the summer maize growing period and climatic conditions under the experimental treatments: **(A)** daily average temperature and rainfall; **(B)** maximum (dotted line) and average (solid line) temperature inside and outside the greenhouse during 8:00-18:00 every day during the treatment period; and **(C)** the light intensity inside and outside the greenhouse.

Supplementary Figure 2 Photo of the greenhouse in the field.

Supplementary Figure 3
**(A)** Location of sampled kernel selection and **(B)** the corresponding location at maturity. The number (white) in the figure is the “kernel number per row,” and the red box is the sampling location.

Supplementary Figure 4 Expression pattern of differentially expressed genes in the kernels under heat treatments (HT).

Supplementary Figure 5 Expression patterns of DEGs involved in photosynthesis pathways of two maize varieties under the control (CK) and heat (HT) treatments. For each gene, the Fragments Per Kilobase of Transcript per Million fragments mapped (FPKM) fold change in maize leaves under the CK and HT conditions was measured.

Supplementary Figure 6 Effects of heat stress on the thousand-kernel weight of maize varieties grown under control (CK) and heat (HT) treatments.

Supplementary Table 1 Sequencing data statistics note.

Supplementary Table 2 Some of the genes used in this study.

Supplementary Table 3 Some of the heat shock genes used in this study.

Supplementary Table 4 List of primer sequences used in this study.
