# Supplementary material for: Heat Stress After Pollination Reduces Kernel Number in Maize by Insufficient Assimilates
Source: Front Genet. 2021 Oct 8;12:728166. doi: 10.3389/fgene.2021.728166 (PMC8532994; doi:10.3389/fgene.2021.728166)

Table S1 Sequencing data statistics note

Samples	Clean reads	Clean bases	GC Content	%≥Q30
XYCKG1	29,708,909	8,888,983,506	54.35%	95.14%
XYCKG2	23,944,362	7,164,200,406	54.45%	95.19%
XYCKG3	23,474,158	7,020,141,474	54.53%	94.73%
XYHTG1	30,785,132	9,212,177,966	54.65%	95.28%
XYHTG2	31,094,707	9,298,303,050	53.88%	95.31%
XYHTG3	30,779,408	9,208,206,250	54.01%	95.26%
ZDCKG1	28,619,405	8,557,422,702	54.99%	95.68%
ZDCKG2	37,429,394	11,201,604,168	54.69%	95.54%
ZDCKG3	28,953,933	8,662,947,934	54.25%	95.73%
ZDHTG1	30,974,093	9,267,139,526	54.53%	95.52%
ZDHTG2	29,277,772	8,755,489,414	54.53%	95.49%
ZDHTG3	27,665,295	8,281,796,356	54.56%	95.20%
XYCKL1	20,182,833	6,034,741,884	57.30%	94.88%
XYCKL2	29,231,611	8,727,540,024	57.21%	94.93%
XYCKL3	26,242,395	7,851,262,276	56.68%	94.97%
XYHTL1	21,548,024	6,442,236,036	57.18%	94.98%
XYHTL2	22,826,335	6,822,630,830	56.39%	94.98%
XYHTL3	23,962,572	7,167,031,848	56.40%	95.07%
ZDCKL1	21,415,741	6,400,827,964	56.65%	95.30%
ZDCKL2	21,877,516	6,541,309,344	56.74%	95.11%
ZDCKL3	23,046,705	6,894,869,672	56.35%	95.38%
ZDHTL1	22,090,091	6,608,384,898	56.66%	95.03%
ZDHTL2	23,145,451	6,912,412,726	55.74%	95.21%
ZDHTL3	23,806,089	7,121,312,872	56.37%	95.00%

Table S2 Some of the genes used in this study

Enzyme	Locus tag	Also known as	
Trehalose-6-phosphate phosphatase	Zm00001d032298	*TRPP1*	GRMZM2G347280
	Zm00001d005658	*TRPP2*	GRMZM2G140078
	Zm00001d006913	*TRPP3*	GRMZM2G117564
	Zm00001d052227	*TRPP4*	GRMZM2G151044
	Zm00001d017502	*TRPP6*	GRMZM2G112830
	Zm00001d018082	*TRPP7*	GRMZM2G055150
	Zm00001d020272	*TRPP8*	GRMZM2G174396
	Zm00001d022193	*TRPP10,*	GRMZM2G014729
	Zm00001d044854	*TRPP11*	GRMZM2G080354
Trehalose-6-phosphate synthase	Zm00001d010755	*TRPS1*	GRMZM2G068943
	Zm00001d028267	*TRPS2*	GRMZM2G099860
	Zm00001d032118	*TRPS3*	GRMZM2G079928
	Zm00001d032311	*TRPS4*	GRMZM2G008226
	Zm00001d005687	*TRPS5*	GRMZM2G527891
	Zm00001d043468	*TRPS6*	GRMZM2G304274
	Zm00001d043469	*TRPS7*	GRMZM2G123277
	Zm00001d050069	*TRPS8*	GRMZM2G007736
	Zm00001d050293	*TRPS9*	GRMZM2G366659
	Zm00001d052060	*TRPS10*	GRMZM2G312521
	Zm00001d018342	*TRPS11*	GRMZM2G122231
	Zm00001d038728	*TRPS12*	GRMZM2G001304
	Zm00001d020396	*TRPS13*	GRMZM2G019183
	Zm00001d008338	*TRPS14*	GRMZM2G416836
	Zm00001d012748	*TRPS15*	GRMZM2G118462
Starch synthase	Zm00001d045261	*SS1*	GRMZM2G129451
	Zm00001d026337	*SS3*	GRMZM2G121612
	Zm00001d002256	*SS2*	GRMZM5G897776
	Zm00001d014150	*SS6*	GRMZM2G126988
	Zm00001d010821	*SS4*	GRMZM2G044744
	Zm00001d000002	*DU*	GRMZM2G141399
	Zm00001d037234	*SU2*	GRMZM2G348551
	Zm00001d051976	*SS5*	GRMZM2G130043
Glucose-1-phosphate adenylyltransferase small subunit	Zm00001d03238	*AGPS1*	GRMZM2G163437
	Zm00001d019266	*AGPII2*	GRMZM2G144002
	Zm00001d050032	*BT2*	GRMZM2G068506
	Zm00001d005546	*AGP1*	GRMZM2G106213
	Zm00001d044129	*SH2*	GRMZM2G429899
Cell wall invertase	Zm00001d003776	*MN1,*	GRMZM2G119689
	Zm00001d016708	*INCW1*	GRMZM2G139300
	Zm00001d025355	*INCW3*	GRMZM2G123633
	Zm00001d001941	*INCW4*	GRMZM2G119941
	Zm00001d025354	*INCW5*	GRMZM2G095725
	Zm00001d001944	*INCW6*	GRMZM2G018692
	Zm00001d001943	*INCW7*	GRMZM2G018716
	Zm00001d041991	*INCW8*	GRMZM2G174249
	Zm00001d014947	*IVr2*	GRMZM2G089836
Ribulose-1,5-bisphosphate carboxylase	Zm00001d004894	*SSu2*	GRMZM2G113033
	Zm00001d052595	*SSu1*	GRMZM2G098520

Table S3 Some of the heat shock genes used in this study

Locus tag	Also known as	
Zm00001d28555	HSP10	AC208204.3_FG006
Zm00001d39936	HSP12	GRMZM2G158232
Zm00001d52855	HSP13	GRMZM2G012631
Zm00001d47548	HSP18a	GRMZM2G404249
Zm00001d8841	HSP18c	GRMZM2G034157
Zm00001d39566	HSP18f	GRMZM2G083810
Zm00001d48073	HSP19	GRMZM2G024718
Zm00001d52194	HSP22	GRMZM2G007729
Zm00001d28408	HSP26	GRMZM2G149647
Zm00001d18298	HSP28	GRMZM2G098167
Zm00001d20898	HSP4	GRMZM2G069651
Zm00001d47799	HSP5	GRMZM2G366532
Zm00001d42922	HSP6	AC209784.3_FG007
Zm00001d10529	HSP7	GRMZM5G802801
Zm00001d30725	HSP70-1	GRMZM2G056039
Zm00001d14358	HSP70-10	GRMZM2G056766
Zm00001d13842	HSP70-12	GRMZM2G153815
Zm00001d37700	HSP70-14	GRMZM2G023232
Zm00001d9950	HSP70-16	GRMZM2G063676
Zm00001d13507	HSP70-17	GRMZM2G111475
Zm00001d33591	HSP70-2	GRMZM2G079668
Zm00001d23802	HSP70-22	GRMZM2G001500
Zm00001d28630	HSP70-6	GRMZM2G428391
Zm00001d51607	HSP70-7	GRMZM2G158093
Zm00001d17809	HSP70-9	GRMZM2G020040
Zm00001d28557	HSP9	GRMZM2G046382
Zm00001d24903	HSP90	GRMZM5G833699

Table S4 List of primer sequences used in this study

Gene ID	Forward primer (5′-3′)	Reverse primer (5′-3′)
GRMZM2G171060	CCAACGGTAGGCTTCAAT	AGAAACTCCACGGCAATAG
GRMZM2G079957	TCCTCGTCTGCCATCAAT	TCCACTCCACTCCACTTG
GRMZM2G115357	GCTCTTCACCAACTCCTT	CCGTCCTTGTCTTCGTAG
GRMZM2G141382	CTCCCACATACCGAACAC	TCCCAGAAGCGAAGATAGT
GRMZM2G077008	CGATCATGGTCTTGGTCCTT	TCCTCCTCCTTCTCCTCTG
GRMZM5G856653	GCCTATACGAGCACTACAA	CTTGACAACAACCGATGAG
GRMZM2G089713	GCCAAGCCTGACCTTATC	AACAGACAGACCTGAGTGA
GRMZM2G129451	ACCTTGTGGTCTCAATCAG	GGATGGTTGCCTTACTCTAA
GRMZM2G001304	CGAATGGCAGAAGCAATT	GCTGTGACTTGATGAGGA

Figure S1 The meteorological data during the summer maize growing period and climatic conditions under the experimental treatments: (a) daily average temperature and rainfall; (b) maximum (dotted line) and average (solid line) temperature inside and outside the greenhouse during 8:00-18:00 every day during the treatment period; and (c) the light intensity inside and outside the greenhouse.

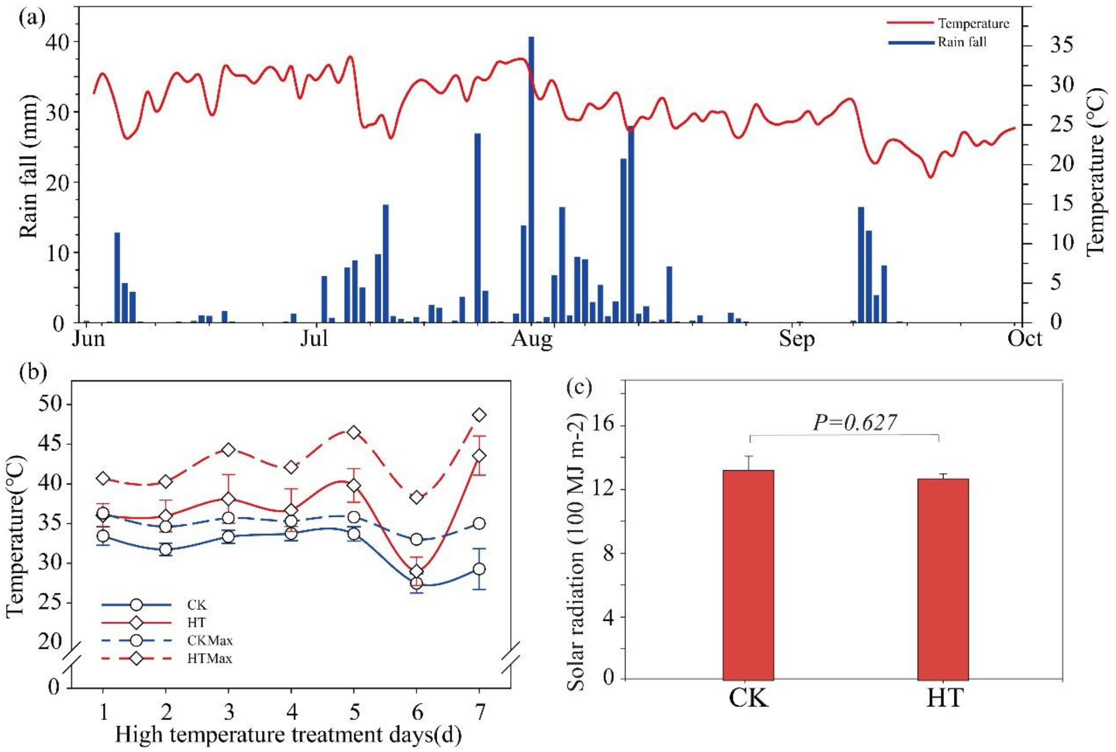

Figure S2 Photo of the greenhouse in the field

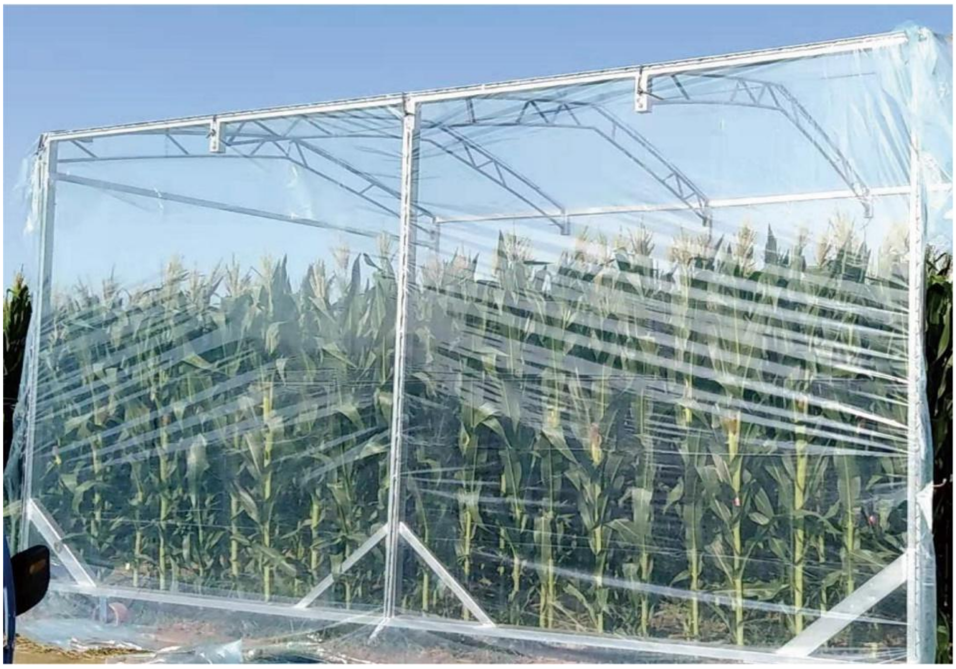

Figure S3 (a) Location of sampled kernel selection and (b) the corresponding location at maturity. The number (white) in the figure is the “kernel number per row”, and the red box is the sampling location.

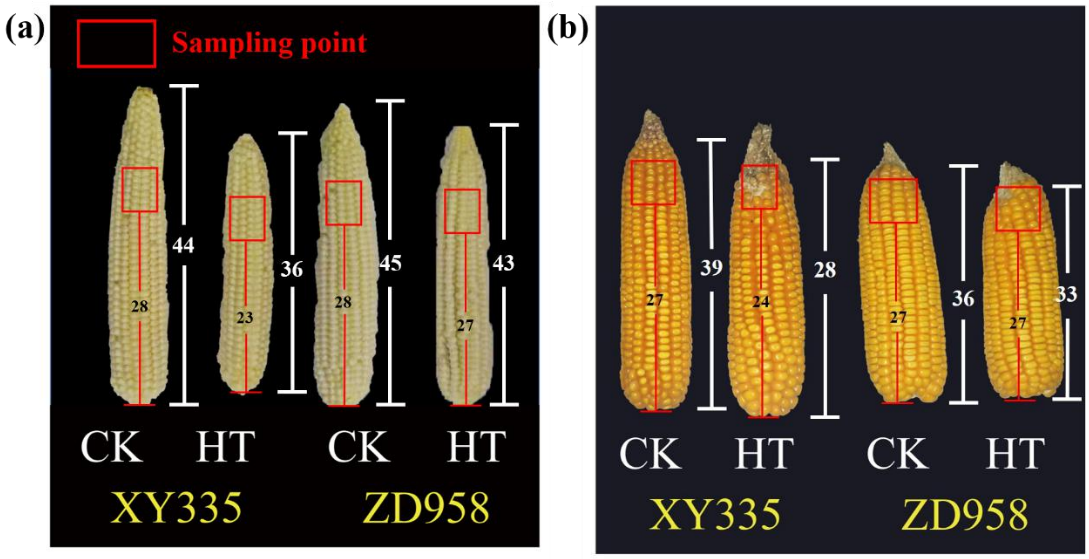

Figure S4 Expression pattern of differentially expressed genes in the kernels under heat treatments (HT).

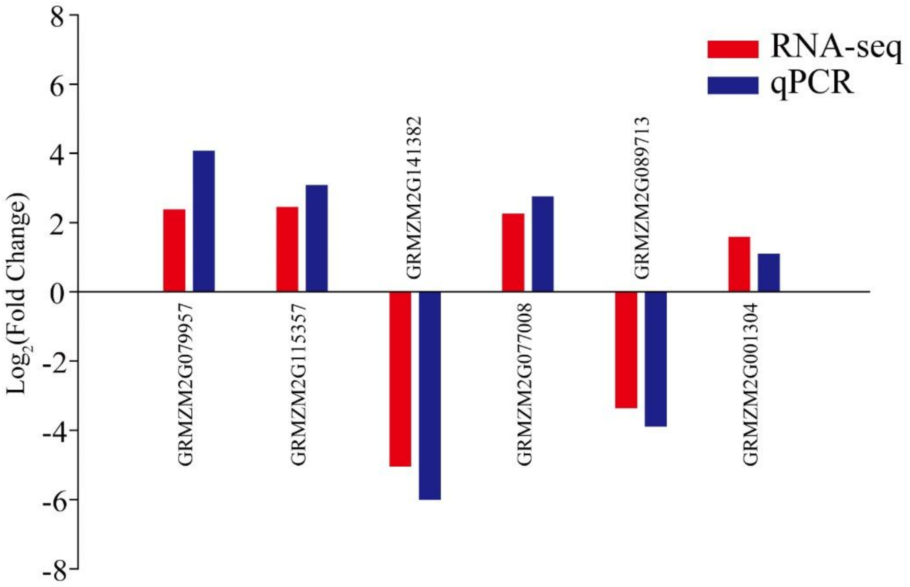

Figure S5 Expression patterns of DEGs involved in photosynthesis pathways of two maize varieties under the control (CK) and heat (HT) treatments. For each gene, the Fragments Per Kilobase of Transcript per Million fragments mapped (FPKM) fold change in maize leaves under the CK and HT conditions was measured.

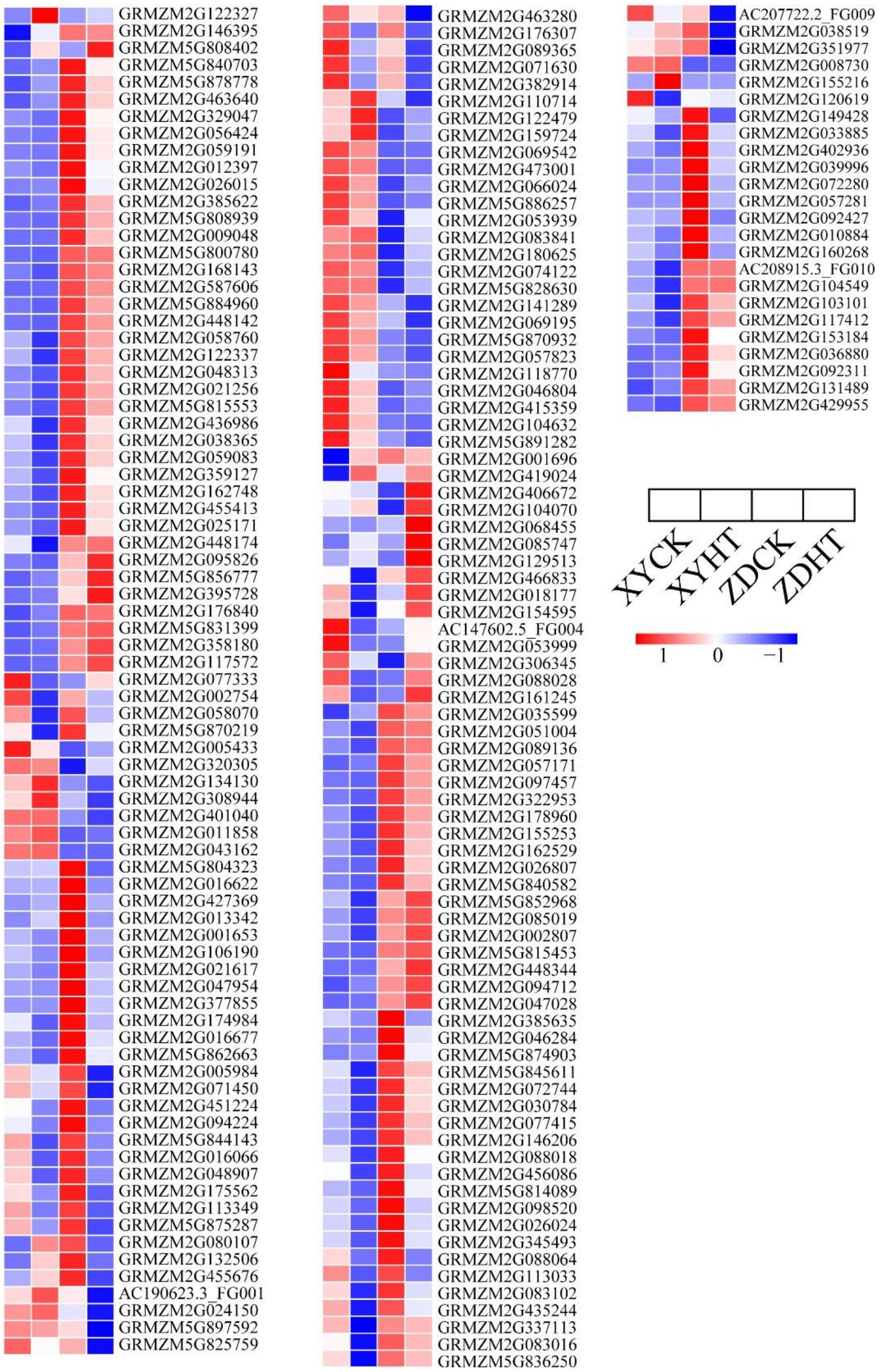

Figure S6 Effects of heat stress on the thousand-kernel weight of maize varieties grown under control (CK) and heat (HT) treatments.

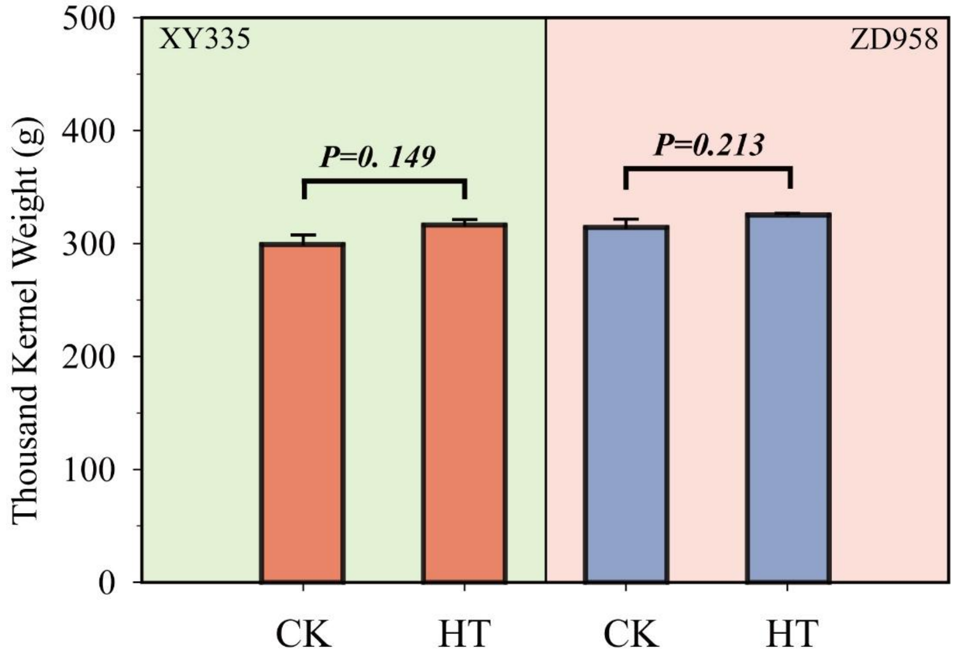

Supplement: Supplementary file 1 [file DataSheet1.pdf]
